# Targeting tumor-associated carbohydrate antigens: a phase I study of a carbohydrate mimetic-peptide vaccine in stage IV breast cancer subjects

**DOI:** 10.18632/oncotarget.21959

**Published:** 2017-10-23

**Authors:** Laura F. Hutchins, Issam Makhoul, Peter D. Emanuel, Angela Pennisi, Eric R. Siegel, Fariba Jousheghany, Xueyan Guo, Anastas D. Pashov, Behjatolah Monzavi-Karbassi, Thomas Kieber-Emmons

**Affiliations:** ^1^ Departments of Medicine, University of Arkansas for Medical Sciences, Little Rock, Arkansas, USA; ^2^ Departments of Biostatistics, University of Arkansas for Medical Sciences, Little Rock, Arkansas, USA; ^3^ Departments of Pathology, University of Arkansas for Medical Sciences, Little Rock, Arkansas, USA; ^4^ Department of Gastroenterology, Third Affiliated Hospital of Xi'an Jiaotong University, Xi'an, Shaanxi, China; ^5^ Stephan Angelov Institute of Microbiology, Bulgarian Academy of Sciences, Sofia, Bulgaria; ^6^ Winthrop P. Rockefeller Cancer Institute, Little Rock, Arkansas, USA

**Keywords:** tumor-associated carbohydrate antigens, cancer vaccine, clinical trial, breast cancer, peptide mimotopes

## Abstract

Tumor-associated carbohydrate antigens (TACAs) support cell survival that could be interrupted by anti-TACA antibodies. Among TACAs that mediate cell survival signals are the neolactoseries antigen Lewis Y (LeY) and the ganglioside GD2. To induce sustained immunity against both LeY and GD2, we developed a carbohydrate mimicking peptide (CMP) as a surrogate pan-immunogen that mimics both. This CMP, referred to as P10s, is the N-terminal half of a peptide vaccine named P10s-PADRE, the C-terminal half of which (PADRE) is a Pan-T-cell epitope. A Phase I dose-escalation trial of P10s-PADRE plus adjuvant *MONTANIDE*™ ISA 51 VG was conducted in subjects with metastatic breast cancer to test 300 and 500 μg/injection in two cohorts of 3 subjects each. Doses of the P10s-PADRE vaccine were administered to research participants subcutaneously on weeks 1, 2, 3, 7 and 19. Antibody responses to P10s, GD2, and LeY were measured by ELISA. The P10s-PADRE vaccine induced antibodies specifically reactive with P10s, LeY and GD2 in all 6 subjects. Serum antibodies displayed Caspase-3-dependent apoptotic functionality against LeY or GD2 expressing breast cancer cell lines. Immunization with the P10s-PADRE vaccine was well-tolerated and induced functional antibodies, and the data suggest potential clinical benefit.

## INTRODUCTION

Aberrant glycosylation is a common phenotypic change of cancer cells, arising from clustered presentation on a cell surface or through modified synthesis [1]. Tumor-Associated Carbohydrate Antigens (TACAs) mediate important signaling effects that underlie their role in tumor biology. Since TACAs are expressed on glycoproteins and glycolipids that regulate multiple cellular/signaling pathways, targeting the proliferative machinery of malignant cells by anti-TACA therapies is an attractive concept to combat cancer. Monoclonal antibodies (mAbs) directed to some TACAs, like those directed to the ganglioside GD2 and the neolactoseries antigen Lewis Y (LeY), are known to inhibit cell signaling that influences cell survival [2–3]. Monoclonal antibodies to these oncotargets are in clinical trials [2] or have been approved [3]. The induction of antibodies to these antigens and related types would therefore be of clinical benefit by providing sustained immunity to inhibit metastatic outgrowth.

Immune tolerance to TACAs has severely restricted the usefulness of most TACAs in immunotherapy development, requiring multiple approaches to direct the immune response [4–6]. We have developed potential TACA-directed vaccines based on carbohydrate-mimetic peptides (CMPs) that induce anti-tumor-reactive humoral [7–9] and cellular [10, 11] responses in mice. These CMPs are Pan-immunogens, developed to induce antibodies reactive with multiple TACAs when immunizing with a single agent [7–10, 12].

We have moved one of these CMPs, with the sequence WRYTAPVHLGDG (referred to as P10s) conjugated to the Pan-T-cell epitope PADRE, into an early-phase clinical trial in Stage IV breast cancer subjects. This CMP was designed to mimic and induce responses to the ganglioside GD2 [9] and the LeY antigen [12, 13]. In particular, LeY has a very restricted tissue distribution because it is a fetal antigen. Historically, LeY was found overexpressed on a large percentage of tumors of epithelial origin including those originating from colon, breast, lung, prostate and ovary [14–17]. The LeY antigen enhances the invasion and metastasis of cancer cells, and its high expression is associated with decreased survival in lymph node-negative breast carcinomas [18]. The LeY antigen is purported to regulate the expression of cell cycle-related factors through ERK/MAPK and PI3K/Akt signaling pathways to promote cell proliferation [19, 20]. LeY expression is therefore considered associated with drug resistance [21].

Likewise, anti-GD2 antibodies can downmodulate PI3K/Akt signaling pathways [22, 23]. As a potential target for anti-tumor immunotherapy, GD2 is ideal due to its high expression on several tumor types and its restricted expression on normal tissue. The National Cancer Institute pilot program for the prioritization of the most important cancer antigens ranks GD2 as #12 out of 75 potential targets for cancer therapy [24]. In fact, GD2 comes in at #6 when considering antigens that are directly targetable in the circulation or on the cell surface. GD2 is expressed on breast and other cancer stem cells [25–27]. The recently demonstrated involvement of the GD2/c-Met axis in estrogen receptor (ER)-negative breast-cancer aggressiveness strongly supports anti-GD2 immunotherapeutic approaches for treatment of breast cancer, especially in subgroups of breast cancer subjects clustering on very aggressive breast cancer subtypes, such as triple-negative and metaplastic variants [28–30]. In addition, gangliosides of the b-series, which includes GD2/GD3, are demonstrated at higher levels in the sera of breast-cancer subjects [31]. Consequently, the induction of antibodies to LeY or GD2 has the potential to interfere with cancer cell-survival signals.

To test the feasibility of inducing proapoptotic antibodies reactive with LeY and GD2, we immunized advanced breast-cancer subjects with the P10s-PADRE vaccine. We characterized tolerability, the feasibility of the immunization schedule, and the humoral response to P10s-PADRE in two cohorts (3 subjects each) at different immunogen doses. The ability of the vaccine-induced antibodies to affect survival of human breast cancer cell lines was examined. Our results provide further insight into the relative importance of inducing humoral responses to TACAs and the potential use of P10s as an immunogen to induce immune responses that could have clinical benefit.

## RESULTS

### Subject characteristics

Sixteen subjects were consented into the study. Six subjects (37.5%) failed screening because of a lack of delayed-type hypersensitivity (DTH) response to two recall antigens, while three subjects (18.8%) failed screening for other reasons. Only one subject declined to continue with the study, and never completed pre-study/screening to determine eligibility. The six subjects who did enroll successfully completed their immunization course of 5 immunizations over 23 weeks – hyperimmunization on weeks 1, 2, and 3, another immunization on week 7, and a boost immunization at week 19. No toxicities were observed. A summary of the subjects’ status and response to the P10s-PADRE vaccination is listed in Tables 1A and 1B.

**Table 1A:** Patient characteristics of all consented subjects

Subject	Eligibility (y/n)	Reason Ineligible	Age	Performance Status (ECOG)	Lines of Prior Endocrine Therapy	Lines of Prior Chemotherapy	ER/PR/Her2 Status
39601	y		77	0	2	1	+/+/−
39603	y		45	0	0	2	+/−/−
39604	y		49	0	0	1	+/−/+
39608	y		50	1	3	1	+/+/−
39609	y		67	0	2	2	+/−/−
39616	y		51	0	0	1	−/−/+
39610	n	withdrew consent	44	0	2	0	+/+/−
39611	n	DTH neg	67		1	1	+/+/−
39614	n	DTH neg	73	0	2	1	+/+/−
39606	n	DTH neg	61		0	3	−/−/−
39605	n	DTH neg	67	0	1	2	+/+/−
39613	n	disease progression	44	0	0	1	−/−/−
39615	n	disease progression	65		2	2	+/+/−
39612	n	DTH neg	62	1	0	1	−/−/−
39607	n	DTH neg	54	0	0	2	−/−/−
39602	n	elevated LFT	61	3	2	0	+/+/−

**Table 1B:** Systemic therapy, clinical responses, and immune responses of enrolled subjects

Dose of peptide (μg) per vaccination	Subject	Standard systemic therapy	Time to Progression, or best RECIST response at last contact if not progressed	Immune responses	
				Immunized serum and plasma binding to MAP (ELISA)	Immunized serum and plasma toxicity on cancer cells
300	39601 (#1)	Denosumab	16 months	Yes	Yes
	39603 (#2)	Carboplatin and Gemcitabine	4 months	Yes	Yes
	39604 (#3)	Vinorelbine and Trastuzumab	12 months	Yes	Yes
500	39608 (#4)	Zometa	8 months	Yes	Yes
	39609 (#5)	Faslodex	SD^‡^ at 54 months	Yes	No†
	39616 (#6)	Trastuzumab	CR^‡^ at 50 months	Yes	Yes

†Due to high background toxicity in preimmune plasma from this subject, no significant increase in plasma toxicity was observed. A close look at the subject's calendar indicated that a Faslodex dose was applied two days before her preimmune blood draw. ‡ SD and CR respectively mean Stable Disease and Complete Response via RECIST v1.1 criteria.

### Immunization with P10s-PADRE induced anti-P10s antibody response

**Figure 1:**
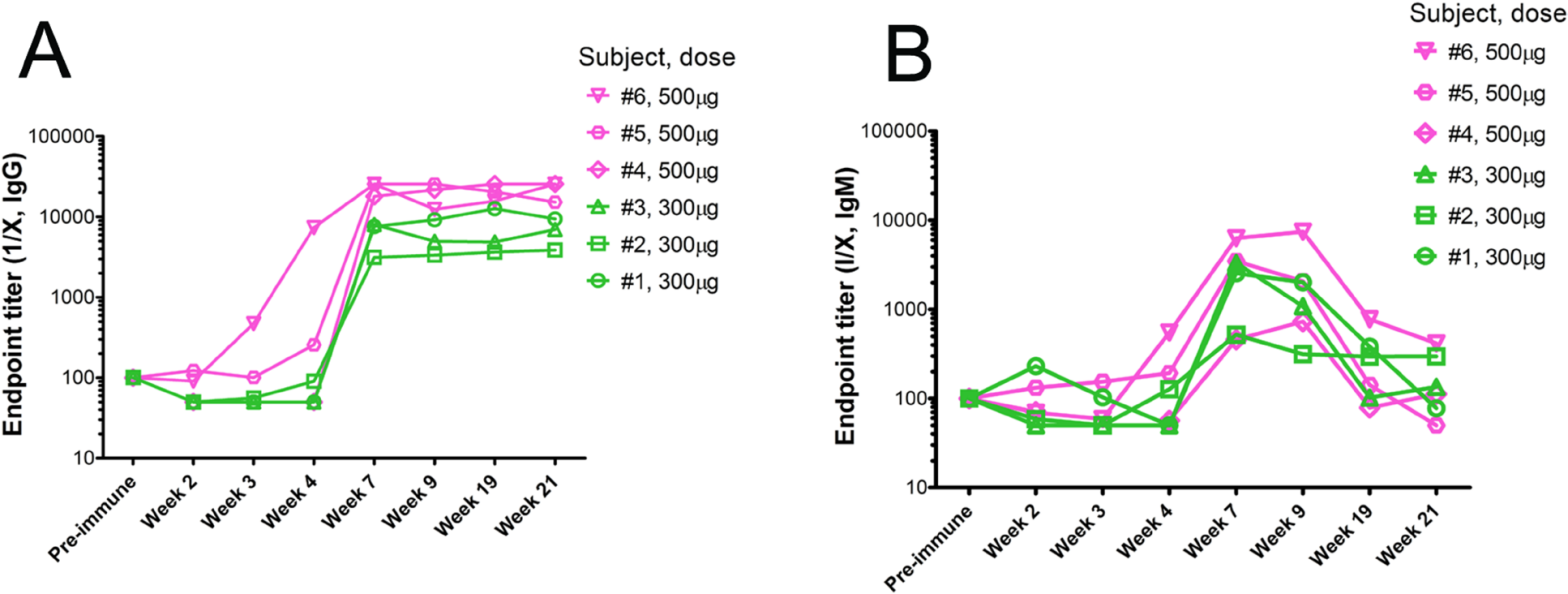
Reactivity of immunized subjects’ serum antibodies against P10s ELISA plates were coated with the multivalent-antigen peptide (MAP) version of P10s, and reactivity of two-fold serial dilutions of sera from the weeks indicated on the horizontal axes was detected by HRP-conjugated anti-human IgG (**A**) and IgM (**B**). Normalized anti-peptide endpoint titers were estimated as described in the Methods section.

The most important feature of the immune response to the P10s-PADRE vaccine is the observation that immunized subjects generated an immune response to the Multiple Antigenic Peptide (MAP) version of P10s, which is free of the PADRE conjugate. The multivalent nature of MAPs allow for a more faithful mimicry of the clustered structures of cell-surface glycans that is extremely useful for testing glycan recognition in solid phase. We observed an increase in IgG and IgM binding to the P10s MAP in subjects’ postimmune sera compared to their preimmune sera (Figure 1). A similar binding pattern to the MAP peptide was observed using plasma samples (Supplementary Figure 1). Anti-P10s IgG and IgM reactivity surged from week 4 to week 7 of the study in all subjects except subject #6, in whom it was already surging at week 4. The increases of IgG at week 7 compared to preimmune ranged from 31 folds (in subject #2) to 256 folds (in subjects #5 and #6), and the titers in subsequent weeks showed little change from their week-7 values. Endpoint titers were tested in yearly follow-up serum samples available from 4 out of 6 subjects vaccinated. The titers dropped but were still considered high a year after final immunization but fell significantly after the second year (Supplementary Table 1). The IgM response was short-lived, as a dramatic decrease was observed by week 19. The cohort immunized with the higher dose of vaccine displayed higher normalized IgG endpoint titers (Figure 1). In nonparametric repeated-measures analysis for IgG-reactive serum, the main effects of dose and week were statistically significant (both *P* values <.0001), but the dose×week interaction was not (*P* = 0.40) (Table 2). The data suggest that 3 immunizations are enough to generate high titers of anti-P10s IgG antibodies in both serum and plasma samples. The data also suggest that 500 μg per immunization may lead to higher antibody titers and a stronger immune response compared to immunization with 300 μg per injection.

**Table 2:** Non-parametric repeated-measures analysis of endpoint titer with Dose as the between-subject effect and Week as the within-subject effect

Source	DF^†^	F^‡^	*P*-value
Dose	1	38.9	< 0.0001
Week	2.93	27.22	< 0.0001
Dose × Week	2.93	0.98	0.40

†:Degrees of Freedom ‡:the ANOVA-Type Statistic of Brunner et al. [33] which approximately follows the central *F*(DF,∞)-distribution.

### P10s-PADRE-immunized serum reacted with both GD2 and LeY antigens

**Figure 2:**
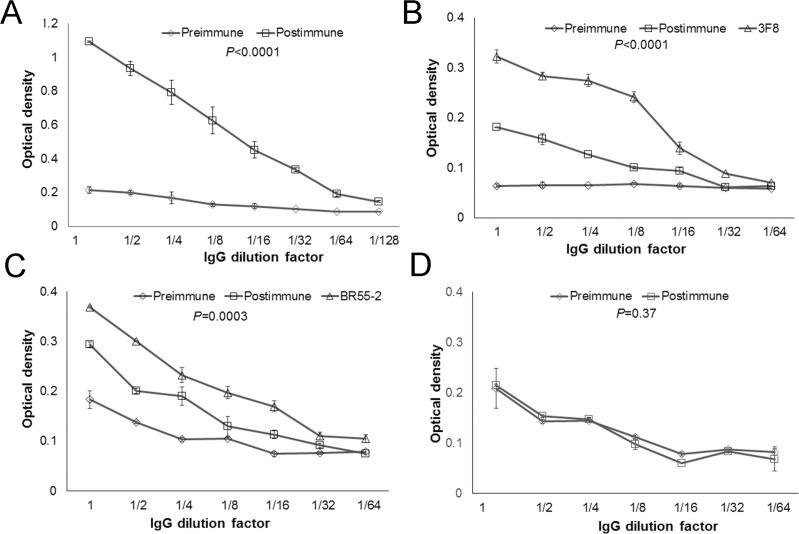
IgG fraction of serum antibodies bind to both GD2 and LeY antigens IgG fraction of pre- and post-immune sera was enriched. ELISA plates then were coated with P10s-MAP (**A**), GD2 (**B**), LeY (**C**), and α2,8-sialic acid (**D**) antigens and serially diluted purified IgG was added to wells. The starting concentration for the IgG fraction was 120 μg/ml. The starting concentration for the mAbs was 20 μg/ml. The binding was visualized after addition of HRP-conjugated anti-human IgG. *P* values comparing pre and postimmune dilution curves for each panel are shown. Symbols ± error bars at each dilution factor denote the Means ± SD of three experiments. Serum samples from Subjects 4, 5 and 6 were tested. We observed similar reactivity pattern for the subjects tested and the data for IgG fraction of serum from subject 6 is shown in this figure.

Since P10s is a mimic of both LeY and GD2, we examined whether postimmune serum binds to these antigens. We observed a similar pattern of reactivity of postimmune serum with both glycans (Table 3). The reactivity patterns of P10s-induced serum antibodies with the glycan antigens in the ELISA assay parallel those from our preclinical studies [9]. The IgG fraction of pre- and postimmune sera was purified and tested for their binding to P10s-MAP, LeY, and GD2 (Figure 2). IgG from immunized serum reacted with P10s-MAP (Figure 2A) and displayed higher binding to both GD2 and LeY glycans than the IgG purified from the preimmune serum (Figure 2B, 2C). No differences between binding of preimmune and postimmune purified IgG to α2,8-sialic acid, used as negative control, was detected (Figure 2D).

**Table 3:** Anti-glycan IgG response in postimmune serum in vaccinated subjects

Subject	Ley	GD2
1	2	4
2	4	8
3	8	4
4	8	8
5	4	4
6	8	4

Fold increase in endpoint titers relative to preimmune serum are shown.

### Immunized serum inhibited the growth of breast cancer cells *in vitro*

The real goal of effective immune therapy that directly targets cancer cells is to show that induced immune responses can result in tumor-cell death. We have shown previously that immunization with a plant lectin-reactive mimotope led to generation of apoptotic antibodies in a mouse model [8]. Anti-LeY and -GD2 antibodies are expected to negatively affect tumor-cell viability through induction of apoptosis and inhibition of growth [19, 23, 32, 33]. Therefore we examined whether serum antibodies from immunized subjects affect viability of human breast cancer cell lines *in vitro*. MDA-MB-231 (triple negative), HCC1954 (Her2-positive), MCF7 (ER-positive,) and ZR-75–1 (ER-positive) cell lines were chosen to examine binding and functionality of serum antibodies. MDA-MB-231 cells are known to express the GD2 ganglioside [28, 34] (Supplementary Figure 2A). HCC1954, MCF-7, and ZR-75–1 cell lines are LeY positive (Supplementary Figure 2B, 2C and 2D).

**Figure 3:**
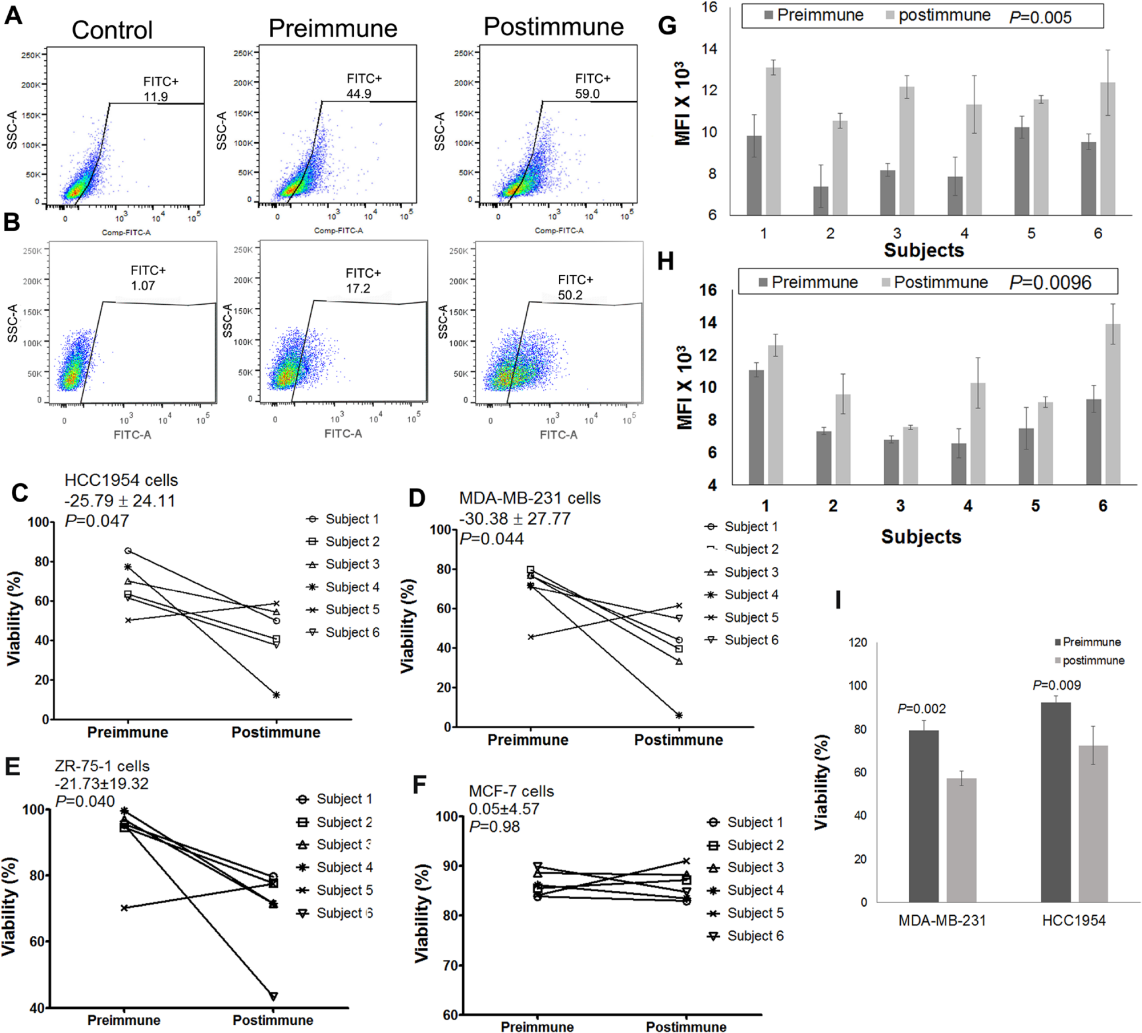
P10s-PADRE induces antibodies that react with cells and negatively affect viability of cells Plasma samples from a representative subject (subject 6) bind to HCC1954 (**A**) and MDA-MB-231 (**B**) cells, Plasma derived from 5 out of 6 subjects negatively affect viability of both HCC1954 (**C**) and MDA-MB-231 (**D**) cell lines. Postimmune antibodies inhibited growth of ZR-75-1 cells (**E**) but did not affect viability of MCF-7 cells (**F**). Means ± SDs of paired differences in cell-line viability are shown in the upper left of panels C, D, E, and F, and are in percentage-point units; paired *t*-test *P* values appear immediately below the viability means ± SDs. Mean fluorescence intensities (MFIs) of all subjects’ pre- and postimmune plasma antibodies binding to MCF-7 (**G**) and ZR-75-1 (**H**) cells are shown. Bar heights ± error bars in panels G, H denote the means ± SDs of MFI; paired *t*-test *P* values for the pre-post difference among all 6 subjects appear after the bar labels. (**I**) Purified IgG fraction of postimmune plasma derived from subject #5 significantly inhibits growth of both HCC1954 and MDA-MB-231 cell lines. Bar heights ± error bars denote the means ± SEM of viability from three experiments with material from subject #5.

Plasma antibodies from P10s-vaccine immunized subjects displayed an increase in binding to both HCC1954 and MDA-MB-231 cell lines with mean ± SD of 892 ± 673 (*P* = 0.023) and 230 ± 140 (*P* = 0.010), respectively. Binding for the representative pre- and postimmune plasmas from subject 6 is demonstrated (Figures 3A, 3B). The effect of P10s-induced antibodies on the viability of each cell line was tested by adding pre- and postimmune plasma to culture medium. Postimmune plasma significantly suppressed the viability of HCC1954, MDA-MB-231, and ZR-75–1 cells *in vitro* (Figures 3C, 3D, 3E). The postimmune-induced decrease in viability among the 6 subjects had an average (standard deviation) of 26% (24%) towards HCC1954, 30% (28%) towards MDA-MB-231, and 22% (19%) towards ZR-75–1, and all decreases were statistically significant (*P* = 0.047, *P* = 0.044, and *P* = 0.040, respectively). In contrast, we observed no significant plasma effect on viability of MCF-7 cells (Figure 3F). However, postimmune plasma from all 6 subjects reacted well with both MCF-7 (Figure 3G) and ZR-75–1 (Figure 3H) cell lines. The data suggest that binding is required but not enough to affect cell viability. We also examined serum functionality on the normal epithelial cell line MCF-10A and did not detect any differences in viability of cells incubated with pre- and postimmune plasma (Supplementary Figure 3), a result that suggests cancer-cell specificity for postimmune antibodies and parallels those from our mouse studies showing a lack of immunopathology on normal epithelial tissues [9, 35].

These data demonstrate that inhibitory effect toward MDA-MB-231, HCC1954, and ZR-75–1 cell lines was > 10 percentage points higher in the postimmune antibodies from 5 of the 6 subjects compared to their preimmune antibodies. Subject 5 displayed an unusually high growth-inhibitory background in her preimmune serum. To understand whether this high background is attributed to serum IgG, we used purified IgG fractions from this particular subject and examined their effect on MDA-MB-231 and HCC1954 cells. Purified IgG fractions behaved as expected, displaying a significant decrease in viability in both cell lines tested upon treatment with postimmune IgG fraction (Figure 3I).

### The effect of P10s-PADRE-induced immune serum on cell viability was caspase-3-dependent

**Figure 4:**
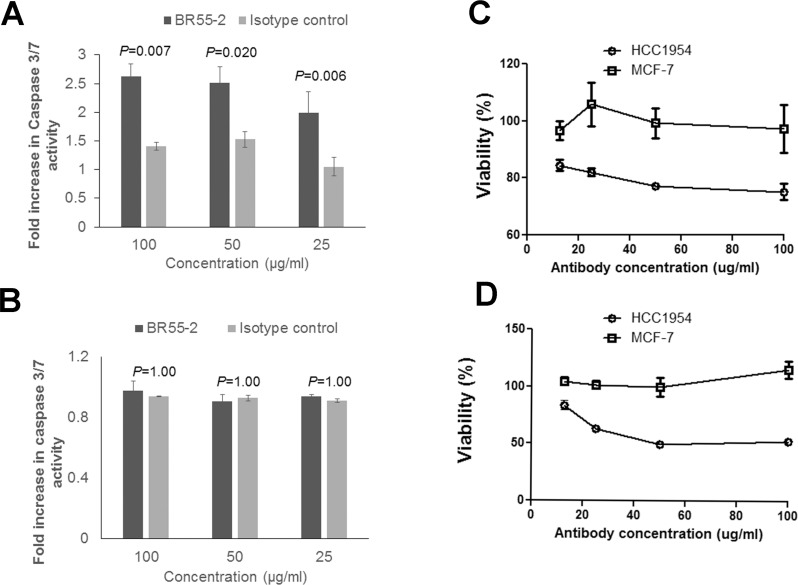
Anti-Lewis Y monoclonal antibody BR55-2 induced caspase-3/7 activation and growth inhibition in HCC1954 HCC1954 (**A**) or MCF-7 (**B**) cells were incubated with various concentrations of BR55-2 and, after 24 hours of incubation, caspase-3 activity was measured. Two-way ANOVA with Bonferroni pos*t*-test was performed and *P* values are shown. Bar heights ± error bars denote means ± SDs of three technical replicates. In separate experiments, the effect of BR55-2 on cell survival was measured after 24 (**C**) and 48 (**D**) hours of incubation using CCK-8 kit. Symbols ± error bars at each tested antibody concentration denote means ± SDs of three technical replicates. BR55-2 negatively affected viability of HCC1954 cells at all concentrations after 24 and 48 hours (*P* < 0.001), while no statistically significant growth inhibition was observed in MCF-7 cells (*P* > 0.05).

As shown above, both MCF-7 and HCC1954 cells overtly express LeY antigen, and postimmune induced antibodies bind to both cell lines. Thus, it is not clear why immune antibodies are effective on HCC1954 but not on MCF-7 cells. MCF-7 cells are known to have a dysfunctional caspase 3, [36] and a lack of growth-inhibitory activity could be due to caspase-3 deficiency in this cell line. Our previously published data [37], through testing for Annexin V-FITC/PI on serum-treated MDA-MB-231 cells, implicated apoptosis in P10s-induced antibody-mediated growth inhibition. If the induction of apoptosis is caspase-3-dependent, then apoptotic antibodies do not affect cells deficient in caspase-3 activity. We next tested the anti-LeY antibody BR55–2's functionality against LeY-positive HCC1954 and MCF-7 cell lines. BR55–2 induced caspase-3/7 activation (Figure 4A) and growth inhibition in HCC1954 (Figure 4C, 4D) but not in MCF-7 cells (Figure 4B, 4C and 4D). The results with BR55–2 on the MCF-7 and HCC1954 cell lines parallel our observations testing functionality of P10s-induced serum antibodies on these cells (Figures 3C, 3F).

**Figure 5:**
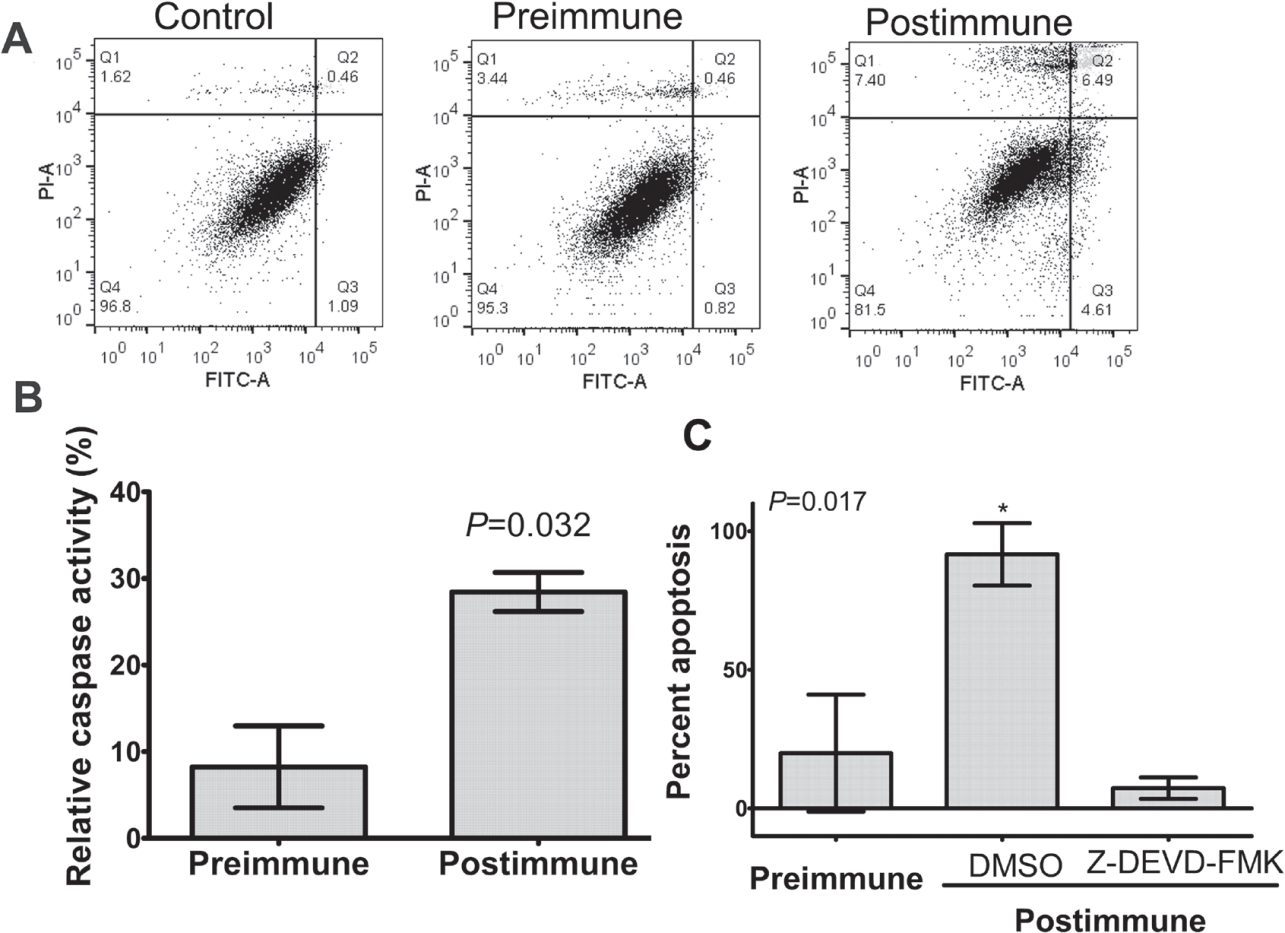
P10s-induced antibodies mediated apoptosis in ZR-75-1 with involvement of caspase 3 (**A**) Cells were incubated with 10% FBS (control) or indicated sera overnight and then harvested and stained with annexin V-FITC (FL1) and propidium iodide (PI-A) using a live/dead assay kit from Invitrogen (Life Technologies, Grand Island, NY). Q3 and Q4 show apoptotic and necrotic cells, respectively¬. (**B**) ZR-75-1 cells were incubated with pre- and postimmune plasma overnight and then relative caspase-3 activity was measured as described in the procedure section. Bar heights ± error bars represent means ± SDs, which were compared by Student's *t*-test and the *P* value is shown. These results are representative of two independent experiments. (**C**) ZR-75-1 cells were preincubated with Caspase-3 Inhibitor Z-DEVD-FMK or DMSO for 30 minutes and then pre- and postimmune plasma samples were added and incubation was continued overnight. Cells were then harvested and stained with Annexin-FITC. Relative positive cell percentage for each treatment was calculated based on cells treated with FBS. Bar heights ± error bars represent means ± SDs, which were compared by one-way ANOVA; the ANOVA *P* value is shown. ^*^, significantly different than preimmune and postimmune plus caspase-inhibitor at *P* < 0.05 using Tukey's post-hoc procedure test. Apoptosis and caspase 3 activity in ZR-75-1 was performed using blood samples from patients 4 and 6 with similar results. The data for subject 6 is shown.

To further investigate the involvement of apoptosis and caspase 3 in immune-serum mediated inhibition of viability we tested subjects’ serum against the ZR-75–1 cell line. ZR-75–1 is a slowly growing ER-positive cell line similar to MCF-7, but expresses both a functional caspase 3 and LeY (Supplementary Figure 2D). Incubation of ZR-75–1 cells with postimmune serum led to annexin-V binding (Figure 5A) and upregulated caspase-3 expression levels (Figure 5B). Inhibition of caspase-3 activity in ZR-75–1 cells in the presence of postimmune serum negated suppressive functionality of antibodies induced by the P10s-PADRE vaccine (Figure 5C).

**Figure 6:**
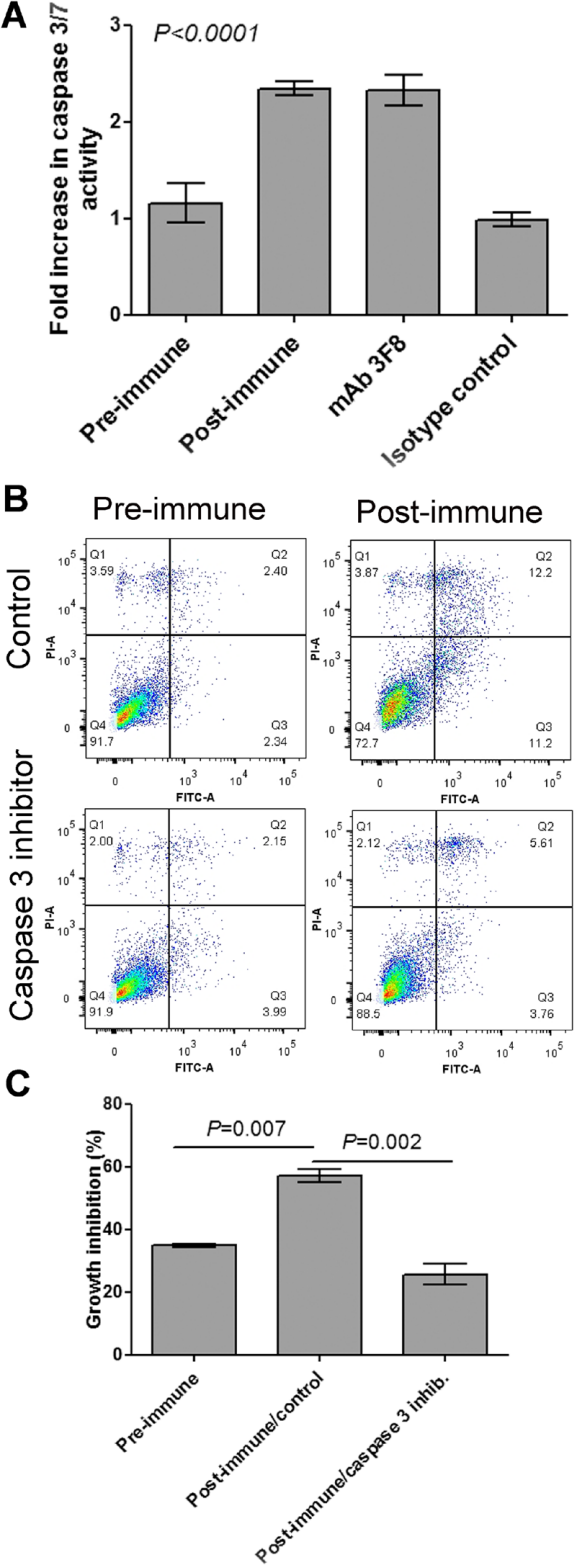
Caspase 3 is involved in post-immune serum-induced growth inhibition of MDA-MB-231 cells (**A**) Cells were incubated with 10% FBS (control), indicated plasma samples, 3F8 mAb or its isotype control for 48 hours and then caspase 3/7 activity was measured. Bar heights and error bars show means and standard deviations, respectively, of three technical replications per treatment. One-way ANOVA with Bonferroni posttest was used to compare means. The *P* value shows significance level comparing either Preimmune with postimmune or mAb with its isotype control. (**B**) MDA-MB-231 cells were preincubated with Caspase-3 Inhibitor Z-DEVD-FMK or DMSO (control) for 2 hours and then pre- and post-immune plasma were added and incubation continued overnight. Cells then were harvested and stained with annexin V-FITC (FL1) and propidium iodide (FL3) using a live/dead assay kit from Invitrogen (Life Technologies, Grand Island, NY). Q3 and Q4 show apoptotic and dead cells, respectively. (**C**) MDA-MB-231 cells were incubated with Pre- and postimmune plasma for 48 hours. Cells treaassigned to treatment with postimmune plasma were preincubated with Caspase-3 Inhibitor or control. Growth inhibition was calculated based on cells treated with FBS. Bar heights and error bars show means and standard deviations, respectively, of three technical replications per treatment. One-way ANOVA with Bonferroni posttest was performed, and resulting *P* values are shown for post-immune/control compared to the other two treatments. Apoptosis and caspase 3 activity in MDA-MB-231 was performed using blood samples from subject 6.

Anti-GD2 monoclonal antibodies are also known to mediate caspase-3/7 cell killing [23]. As a further positive control, caspase-3/7 activity was measured in MDA-MB-231 cells incubated with anti-GD2 mAb 3F8 and sera collected from immunized subjects. Incubation of MDA-MB-231 cells with immunized serum upregulated caspase-3 expression levels along with the anti-GD2 mAb 3F8, but not with an isotype control antibody (Figure 6A). Abrogation of postimmune serum-induced apoptosis and growth inhibition of MDA-MB-231 cells by caspase-3 inhibitor further supports the conclusion that the proapoptotic functionality of antibodies induced by P10s depends on caspase-3 (Figures 6B, 6C).

### P10s-PADRE-induced immune serum was effective in 3D spheroid culture and sensitized spheroids to Paclitaxel treatment

**Figure 7:**
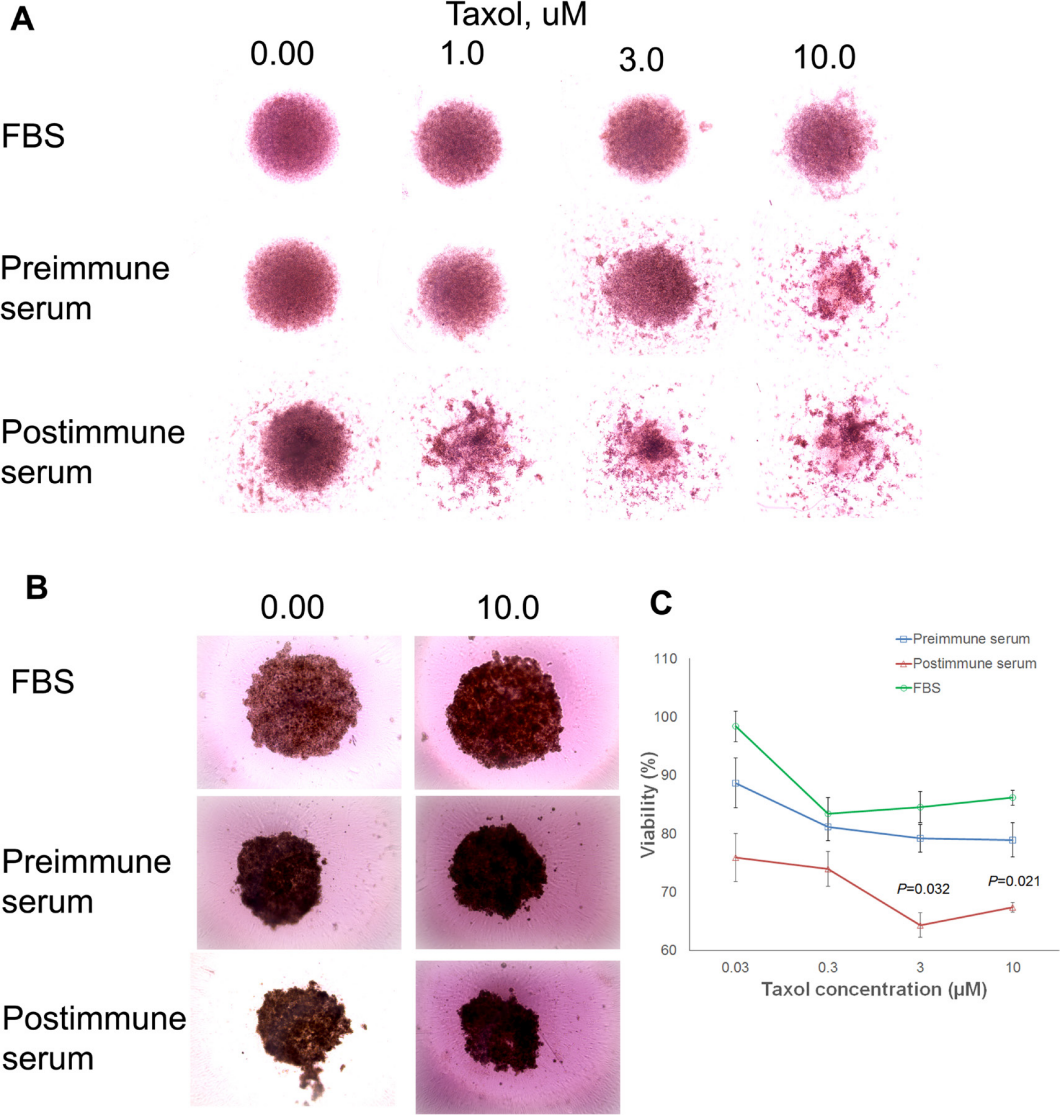
Immune serum enhancement of taxane effect Preincubation of MDA-MB-231 (**A**) and ZR-75–1 (**B**) 3D culture with post-immune serum collected from subject 6 enhanced the effect of taxol on the spheroids. Cells were cultured in ultra-low adhesion round-bottom plates to force spheroid formation. 3000 cells were cultured in RPMI complete medium for 3 days. Pre- and post-immune sera then were added and cells were incubated for 24 h, when fresh medium containing various concentrations of Taxol was added. After another two days of incubation, spheroids were photographed using EVOS™ XL Core cell imaging system at 10x magnification. Adobe Photoshop then was used to compile individual images of various treatments in the figure as demonstrated. (**C**) At the end of the treatment period of ZR-75-–1 cells, alamarBlue^®^ assay was used to measure viability of cells in replicated wells, and percent viability was calculated relative to non-treated cells grown in normal medium supplemented with FBS. Symbols ± error bars at each taxol concentration denote means ± SDs of three technical replications. The experiment was repeated two times with similar results. *P* values suggesting statistically significant differences between pre- and postimmune sera at 3 and 10 uM concentrations of taxol are shown.

To examine the growth-inhibitory properties of the P10s-induced antibodies in a more relevant setting, spheroid cultures were formed, and the impact of pre- and postimmune serum alone and in combination with Paclitaxel on spheroid cultures of MDA-MB-231 and ZR-75–1 cells were detected (Figure 7). 3D-cultured cells that form dense multicellular spheroids may be better than 2D-cultured cells in simulating important tumor characteristics *in vivo*, namely hypoxia, dormancy, anti-apoptotic features and their resulting drug resistance. We observed that postimmune serum disrupted spheroids of MDA-MB-231 cells and enhanced the effect of Paclitaxel treatment (Figure 7A). These results parallel our 2D studies with Docetaxel [37]. In ZR-75–1 cells the spheroids shrink in size upon combination of paclitaxel with immunized serum (Figure 7B) resulting in less viable cells (Figure 7C).

## DISCUSSION

It is well known that TACA clustering or density has a substantial effect on recognition by antibodies [38, 39] and that the characteristics of carbohydrate epitopes on the surface of tumor cells is a governing factor for immune recognition and antibody-mediated targeting of tumor-associated carbohydrate antigens in cancer immunotherapy [40]. Likewise, density-dependent lectin-glycan interactions is a paradigm for conditional regulation by posttranslational modifications [41]. Antibodies reactive with TACAs are suggested to be selective for clustered TACA even in the presence of normal tissue [9, 35, 42]. Consequently, TACAs resulting either from restricted distribution on normal cell surface, incomplete synthesis or neosynthesis accumulate in high density or clustered configuration (possibly in novel conformations relative to glyans expressed on normal tissue) at the tumor cell surface selectively results in a lack of immune mediated tissue damage despite that antibodies can bind to “normal” tissue [9, 35].

Interfering with signaling mechanisms associated with TACA expression can stop the growth of tumors, and prevent the development of metastases [43]. TACA-reactive antibodies are known to be proapoptotic both as monoclonal antibodies and as part of immune surveillance [44–46]. Both the LeY and ganglioside GD2 antigens can regulate signaling processes in several different ways that lends themselves to the progression of cancer [47–49]. Taking advantage of the polyspecific nature of antibodies, we developed P10s as a Pan-immunogen, reacting with multiple anti-TACA antibodies and lectins [45]. Therefore, the induction of polyreactive, proapoptotic antibodies would be a beneficial feature in cancer-immunotherapy approaches. The induction of such antibodies can provide for longer-term survival of cancer subjects, as such antibodies are clinically correlated with long-term survival [50]. Consequently, we developed P10s for clinical testing of vaccination of high-risk breast cancer subjects [9].

The vaccine was administered to subjects who met all eligibility criteria including the ability to mount a response to two recall antigens by skin testing. The rationale for this latter criterion was to select those subjects who have an active immune system capable of reacting to antigenic challenges. We employed a conservative 3 + 3 dose-escalation design for this study, guided by the principle that this design is prudent when considering drugs that are associated with apoptosis [51, 52]. Six subjects met all criteria. All subjects enrolled on the study showed evidence of reactivity to the P10s-PADRE vaccine after immunization. We conclude that immunization with P10s-PADRE is feasible based on the high rate (100%) of subjects who received all five immunizations on schedule.

P10s immunization induced antibodies reactive with P10s and specific for both LeY and GD2. No discernable binding was observed for the irrelevant α2–8 glycan. Assuming that anti-glycan antibodies in postimmune serum are of the same affinity as their corresponding monoclonal antibodies BR55–2 and 3F8, the concentration of anti-LeY and anti-GD2 antibodies is estimated as 1.8 μg/ml and 2.5 μg/ml, respectively, of the total IgG fraction. Collectively, these data suggest that vaccination with P10s-PADRE induces a cross-reactive IgG response to both LeY and GD2 antigens as intended and demonstrating functional capability against tumor cells at relatively low single-digit μg/mL serum antibody concentrations. Our results parallel our preclinical studies where we have shown that the induction of low titers of serum antibodies upon CMP immunization is sufficient to inhibit tumor growth in therapeutic and prophylactic murine tumor models [7, 8, 53].

Evaluation of the antibody response to P10s suggests that the 500 μg dose may produce higher titers of anti-P10s IgG than the 300 μg dose, although the small cohort sizes and lack of randomization preclude firmer conclusions regarding a dose effect. The titer analysis further suggests that only 3 immunizations are sufficient to generate an effective immune response with IgG titers sustained from week 7. Moreover, since no safety concerns were observed in subjects at the 500 μg dose, but concerns exist about excessive adjuvant-related skin reaction with higher doses, it is deemed prudent to recommend 500 μg per injection in future studies.

While P10s generated antibodies in all subjects, growth-inhibitory activity of postimmune antibodies was significantly higher than that of preimmune antibodies in 5 of the 6 subjects on three human breast cancer cell lines, with two of the cell lines considered representative of the extreme resistance to standard systemic therapy. MDA-MB-231 cells are representative of basal-like carcinoma, whereas HCC1954 cells express HER2, but nonetheless have a *de novo* resistance to Trastuzumab. Moreover, for the 6 subjects as a whole, the average decrease in viability was statistically significant for both cell lines. Preimmune plasma from subject 5 displayed high levels of growth inhibition toward both cell lines. Testing the growth-inhibitory effect of purified IgG for this particular patient indicates a lack of involvement of IgG antibodies in the high background of crude preimmune plasma. This subject was treated with Faslodex^®^ (Fulvestrant) two days before her preimmune blood draw. Fulvestrant is a selective ER down-regulator with a long half-life that can induce cell death in ER-positive breast cancer cells [54, 55]. Fulvestrant also inhibits growth of triple-negative breast cancer cell lines [56]. Therefore, the high growth-inhibitory function of subject 5's preimmune plasma antibodies could be due to the residual effect of her drug treatment skewing the results

We observed some direct clinical benefit in one of our subjects with metastatic lesions as evaluated before and after vaccine treatment. This subject (39604) has been previously reported [37]. She had Her2-positive cancer with brain metastases, one of which was resected as part of her standard care; it was cystic and histologically negative for cancer. This subject has had progression including one additional brain metastasis (6 years after vaccine), but is alive and continuing standard therapy. A second subject (39616), also Her2-positive, achieved a complete response and is remaining in remission 4 years off of all therapy. A third patient (39609) has continued the same endocrine therapy with stable disease for 54 months post vaccine. A fourth subject (39608) progressed after 8 months, but is still alive and stable. We speculate that some degree of synergism might exist between the vaccine-induced antibodies and some systemic agents [37]. We observed that pre-incubation with postimmune serum enhanced paclitaxel-induced cell damage in 3D cultures. Multicellular tumor spheroids of cell lines mimic avascular tumor areas characterized by the establishment of diffusion gradients, reduced proliferation rates and increased drug resistance [57–59]. Manipulation of apoptosis has emerged as a new therapeutic strategy to eliminate cancer cells. Our results indicate that P10s-induced immune serum potentiates chemosensitivity to taxanes. Paclitaxel (Taxol) is known to arrest cells in mitosis through microtubule stabilization and to induce apoptosis [60, 61]. Paclitaxel is found to exhibit cytotoxic and antitumor activity by inducing apoptosis via caspase-3 activation [62, 63]. Some studies suggest that GD2-specific monoclonal antibodies induce apoptosis and enhance cytotoxicity of chemotherapeutic drugs [33]. GD2 ganglioside-specific antibody treatment downregulates the PI3K/Akt/mTOR signaling network [22]. Likewise, the LeY antigen increases tumor cell drug resistance [64, 65]. An in-depth understanding of signaling transduction pathways that overcome chemotherapy resistance will provide a basis for increasing chemosensitivity and developing new chemotherapies.

The evidence in this study serves as a proof of principle for immunization using P10s-PADRE to induce antibodies that are proapoptotic against human breast cancer cells. While not a study endpoint, it is worth noting that the overall survival among the subjects treated with the P10s vaccine had a mean ± SE of 52 ± 9 months (median not reached). A randomized clinical trial is required to assess the efficacy of P10s in treating this patient population. It also speaks to the idea of other means of targeting TACA expressing tumor cells. CAR-T cells, for example, have been developed to target glycoforms of MUC1 [66] and against the Chondroitin sulfate proteoglycan isoforms [67]. CAR-T cells have been developed against GD2 [68–70] and LeY [71, 72]. Consequently, multiple immunotherapy approaches targeting TACAs may finally find their way to clinical success.

## MATERIALS AND METHODS

### Experimental design

### Study design

Between July 2011 and December 2013, we conducted a Phase I dose-escalation trial to assess the safety and tolerability of the CMP vaccine, P10s-PADRE, in subjects with Stage IV breast cancer. This trial utilized the conventional 3 + 3 cohort design for dose-escalation studies [73], was approved by the Institutional Review Board (IRB) of the University of Arkansas for Medical Sciences (UAMS), and was registered with the NIH clinical-trials registry at http://clinicaltrials.gov. Women 18 years of age or older, of all races, with histologically or cytologically confirmed stage IV breast cancer were eligible, and subjects were enrolled after providing written informed consent. Disease staging was done according to the American Joint Commission on Cancer (AJCC), sixth edition.

The following eligibility criteria were used: The cancer may be newly diagnosed metastatic or relapsed after primary or adjuvant therapy, and must not have required a treatment change for 2 months. Treatments with anti-estrogen therapy or chemotherapy were allowed. The chemotherapy regimen could not contain steroids in the pre- or post-supportive-care medications. Additional eligibility criteria included: an Eastern Cooperative Oncology Group performance status of 0–1 and adequate organ function (white blood cell count ≥ 3,000/ mm^3^, hemoglobin ≥ 8.0 g/dL, platelets ≥ 100,000/mm^3^ within 2 weeks prior to registration, total bilirubin ≤ 3.0 mg/dL, aspartate aminotransferase ≤ 200 IU/L, alanine aminotransferase ≤ 200 IU/L, and serum creatinine ≤ 1.5 mg/dL). Subjects had to be immunocompetent as measured by responsiveness to skin-test challenges with recall antigens from Trichophyton and Candida.

The following exclusion criteria were applied: known brain metastasis; pregnancy or lactation; known history of HIV infection; clinically serious infection; severe cardiac insufficiency; other active malignancy; history of organ allograft; immunodeficiency or history of splenectomy; concurrent treatment with steroids or immunosuppressive agents; and unsuitability for the trial, based on clinical judgment.

### Vaccine preparation and immunization schedule

P10s (WRYTAPVHLGDG) was covalently attached to the Pan-T-cell peptide PADRE (dAKchAVAAWTLKAAdA: AmbioPharm, Inc., North Augusta, SC, USA). The peptide vaccine was administered in liquid form, emulsified with the adjuvant Montanide ISA-51VG, (SEPPIC, Inc., Fairfield, NJ, USA), by subcutaneous (SC) injections on 5 separate occasions during Weeks 1, 2, 3, 7, and 19. The peptide was synthesized according to Good Manufacturing Practice guidelines.

### Treatment schema

Subjects were treated in groups of three, the first at the initial dose of 300 μg P10s-PADRE per injection. If there were no dose-limiting toxicities (DLTs) in this first group, we planned to escalate to the final dose of 500 μg P10s-PADRE per injection, and treat the next group at this final dose. If there were two or three DLTs in the first group, we planned to de-escalate to a safety dose of 100 μg per injection, and treat three subjects at this safety dose. If there were exactly one DLT in the first group, we planned to vaccinate three more subjects at the initial dose, and escalate to the final dose only if no additional DLTs were observed. All subjects were included in the safety assessment.

### Endpoint variables

Assessment of safety was the primary endpoint. The secondary endpoints were anti-P10s antibody titers, antibody binding to breast cancer cells, and toxicity towards breast cancer cells.

### Adverse event monitoring

Adverse events were assessed using the NCI Common Terminology Criteria for Adverse Events (CTCAE), Version 4.0.

### ELISA assay and IgG purification

ELISA was used to determine the titer of anti-P10s antibodies in the serum and plasma of subjects before and after vaccination as described earlier [37]. The anti-P10s-MAP endpoint titers were estimated from absorbance-versus-dilution curves by linear regression as previously described [37]. Briefly, the intercept of the regression line for each subject's preimmune serum was the value used as the absorbance cutoff for determining end-point titer for that subject, and the dilution where each sample's regression line crossed the subject's absorbance cutoff was defined to be the sample's end-point titer. Extrapolation beyond one dilution below the minimum or above the maximum actual dilutions was not allowed.

To assess binding of vaccine-induced antibodies to GD2 or LeY, plates were coated with ganglioside GD2 (3 μg/ml, Sigma- Aldrich, Saint Louis, MO) or LeY-PAA (Glycotech, Gaithersburg, MD). After blocking, sera were added and incubated for 2 h at 37°C and then after washing, the wells were incubated with goat anti-human IgG (Sigma-Aldrich) for 1 h at 37°C. Binding was visualized and plates were read using an ELISA reader at 450 nM. To determine anti-glycan endpoint titers, the mean background (preimmune) absorbance plus 3 standard deviations was used as the cutoff absorbance. Then, mean absorbance was calculated from duplicates for pre- and postimmune serum dilution, and the highest dilution with an absorbance above the cutoff absorbance was used to determine the endpoint titer. The fold change in endpoint titer was then calculated by dividing the endpoint titer for postimmune to the endpoint titer for preimmune sera [9]. IgG fraction of pre- and post-immune plasma or sera was enriched using Nab™ Protein G Spin Kit and Zeba ™ desalting columns (Thermo Scientific, Rockford, IL). Anti-GD2 and -LeY reactivity of purified IgG was examined by ELISA as described above. Serum concentrations of anti-LeY and anti-GD2 antibodies were determined from binding-curve shifts. Briefly, binding-curve shifts were calculated from the log-log plots of amount of bound antibodies vs immunoglobulin concentration by minimizing the mean square difference between the control curve (mAb) and the theoretical test curve using the slope and intercept of each theoretical curve (serum IgG) and concentration values corrected by the shift value. The calculation and optimization were done in Excel using Solver.

### Cell lines, tissue culture and MAbs

Human breast cancer cell lines were purchased from ATCC (Manassas, VA) and most cell experiments were performed within 6 months after their receipt. However, after the completion of experiments outlined in this paper, the identity of all cell lines used in this study was confirmed by the Human Cell Line Authentication test (Genetica DNA Laboratories, Burlington, NC). Cells were cultured in a base medium supplemented with 10% heat-inactivated fetal bovine serum (Life Technologies), 50 units/mL penicillin, and 50 μg/mL streptomycin. The base medium for MDA-MB-231 was DMEM, for HCC1954 was RPMI, and for MCF-7 cells was insulin-supplemented MEM (all from Fisher Scientific, Pittsburgh, PA). MCF-10A cells were grown in MEGM complete medium (Lonza, Basel, Switzerland) that was replaced by RPMI containing 10% FBS 24 hours prior to treatment with blood samples. Serum, plasma, purified IgG or mAbs were used to treat cells. MAbs used in our studies include the LeY reactive MAb BR55–2 (a gift from Zenon Steplewski [74]), 14G2a (BD Biosciences, San Jose, CA) and 3F8 (a gift from Dr. Ni-Kong V. Cheung, Memorial Sloan Kettering Cancer Center, New York, NY).

### Flow cytometry

Binding of serum or plasma antibodies to breast cancer cell lines was determined as described earlier [37]. Briefly, cells were harvested with an enzyme-free cell-dissociation buffer (Life Technolgies, Grand Island, NY), washed with flow-cytometry buffer (PBS containing 1% BSA and 0.1% sodium azide) and incubated with pre-immune and post-immune samples or mAbs in the same buffer. Binding was visualized with a FITC-conjugated goat anti-human (BD Biosciences, San Jose, CA) or mouse IgG (Sigma-Aldrich).

### Tumor-cell growth-inhibition and cell-toxicity assays

The functional effect of immunized blood samples against breast cancer cell lines was performed by cell toxicity and growth inhibition assays. The cytotoxicity was assayed as previously described [37]. Briefly, 5 × 10^4^ (24-well plate) cells were seeded in medium containing 10% FBS. After 24 hours of incubation, the medium was refreshed with media containing 10% preimmune or postimmune serum or plasma, based on availability, as indicated in the figure legends. The medium in control wells contained 10% FBS. Twenty-four hours after addition of blood samples, supernatants were removed, and live cells were fixed, stained with Crystal violet, and counted. Percent viability was calculated as the percentage of cells surviving in the treated wells relative to the cells in control wells. Cell Counting Kit-8 (CCK-8, Dojindo Molecular Tech. Gaithersburg, MD) was also used to measure viability. All samples were assayed in triplicate. Images were taken using EVOS™ XL Core cell imaging system (Thermo Fisher Scientific, Waltham, MA) at 10X magnification.

The growth-inhibitory role of serum antibodies against MDA-MB-231 and ZR-75–1 cells was assayed in a 3-D culture. 3000 cells were seeded in Corning^®^ round-bottom ultra-low attachment 96-well plates (Corning Inc., Lowell, MA) and were allowed to grow for 2 to 3 days to generate spheroids. Pre- and postimmune sera were then added into wells as described above. The cells were left to grow for 24 hours when the medium was refreshed using medium containing various concentrations of Taxol. Cells were then left to grow for another 48 hours, after which pictures were taken and viability was determined using alamarBlue^®^ cell viability assay (Thermo Fisher Scientific Inc., Waltham, MI) according to the manufacturer's instructions. Fluorescence intensity was measured (Microplate Fluorescence Reader (Bio-Tek Instruments, Winooski, VT) and percent viability relative to the wells with FBS was quantified. Images were taken using EVOS™ XL Core cell imaging system at 10X magnification.

### Apoptosis assay and Caspase-3 activity

Apoptosis was determined using FITC-conjugated Annexin V (Life Technologies, Grand Island, NY) by flow cytometry as described earlier [37]. Caspase-3 activity was measured using Apo-ONE^®^ Homogeneous Caspase-3/7 Assay (Promega, Madison, WI), following manufacturer's instructions. Briefly, cells were seeded in wells (1×10^4^ cells/ well) of a 96-well plate in complete media overnight. Media were replaced the next day with media supplemented with 10% of either FBS (control), preimmune, or postimmune serum or plasma. Alternatively, mAbs were added in medium containing 5% FBS. Cells were incubated at 37°C for an additional 8 to 48 hours, then Apo-ONE^®^ Caspase-3/7 Reagent was added to wells in a 1:1-volume ratio. Plates were then gently shaken at 500 rpm for 1 min and incubated at room temperature for 1–2 hours. Caspase activity was quantified using a fluorescence reader with a 490-nm excitation filter and 520-nm emission filter.

To inhibit caspase activity in the presence of serum or plasma samples, cells were seeded in complete medium, and after overnight culture, supernatant was removed and replaced with serum-free medium. The caspase-3 inhibitor Z-DEVD-FMK (R&D Systems, Minneapolis, MN) was added to wells at a final concentration of 50μm/ml. Z-DEVD-FMK is an effective irreversible caspase inhibitor with no cytotoxic effects. DMSO was added to the control wells. Cells were incubated with caspase inhibitor at 37°C for 2 hours, then preimmune and postimmune samples were added, and the incubation continued.

### Statistical assessments

The resulting absorbance-versus-dilution curves derived from anti-peptide ELISA assays were used to estimate normalized endpoint titers as previously described [37]. SAS 9.3 (The SAS Institute, Cary, NC) was used to calculate the endpoint titers of anti-P10s antibodies and analyze them for changes with respect to time, dose cohort, and their interaction using the nonparametric repeated-measures method of Brunner *et al*. [75] and in particular, using the recommended ANOVA-type statistics from this method. Excel 2010 (Microsoft Corporation, Redmond, WA) was used to evaluate the effect of vaccination on plasma cytotoxicity towards cell lines by one-sample *t*-test on paired differences. To compare the binding of purified IgG fraction of pre and postimmune sera to the peptide and carbohydrates, the slopes of the dilution curves were calculated and compared using regression analysis performed by GraphPad Prism version 5.00 for Windows (GraphPad Software, San Diego, CA).

## CONCLUSIONS

This early-phase clinical trial of a CMP vaccine shows that immunization is feasible and safe, and that immunization with P10s-PADRE vaccine induces a potentially beneficial immunologic response with the induction of proapoptotic antibodies.

## SUPPLEMENTARY MATERIALS FIGURES AND TABLE



## Abbreviations

ANOVA: analysis of variance
CMP: carbohydrate mimicking peptide
DLT: dose-limiting toxicities
GD2: ganglioside disialic 2
GM2 and GM3: ganglioside monosialic 2 and 3
HER2: Her2/neu proto-oncogene neu
IgG: immunoglobulin G
IgM: immunoglobulin
IRB: Institutional Review Board
LeY: Lewis Y antigen
mAb: monoclonal antibody
PADRE: pan DR T helper epitope
SC: subcutaneous
SE: standard error
TACA: tumor associated carbohydrate antigens
UAMS: University of Arkansas for Medical Sciences
