# Supplementary material for: Targeting tumor-associated carbohydrate antigens: a phase I study of a carbohydrate mimetic-peptide vaccine in stage IV breast cancer subjects
Source: Oncotarget. 2017 Oct 23;8(58):99161–78. doi: 10.18632/oncotarget.21959 (PMC5716801; doi:10.18632/oncotarget.21959)
Supplement: Supplementary file 1 [file oncotarget-08-99161-s001.pdf]

Targeting tumor-associated carbohydrate antigens: a phase I study of a carbohydrate mimetic-peptide vaccine in stage IV breast cancer subjects

SUPPLEMENTARY MATERIALS

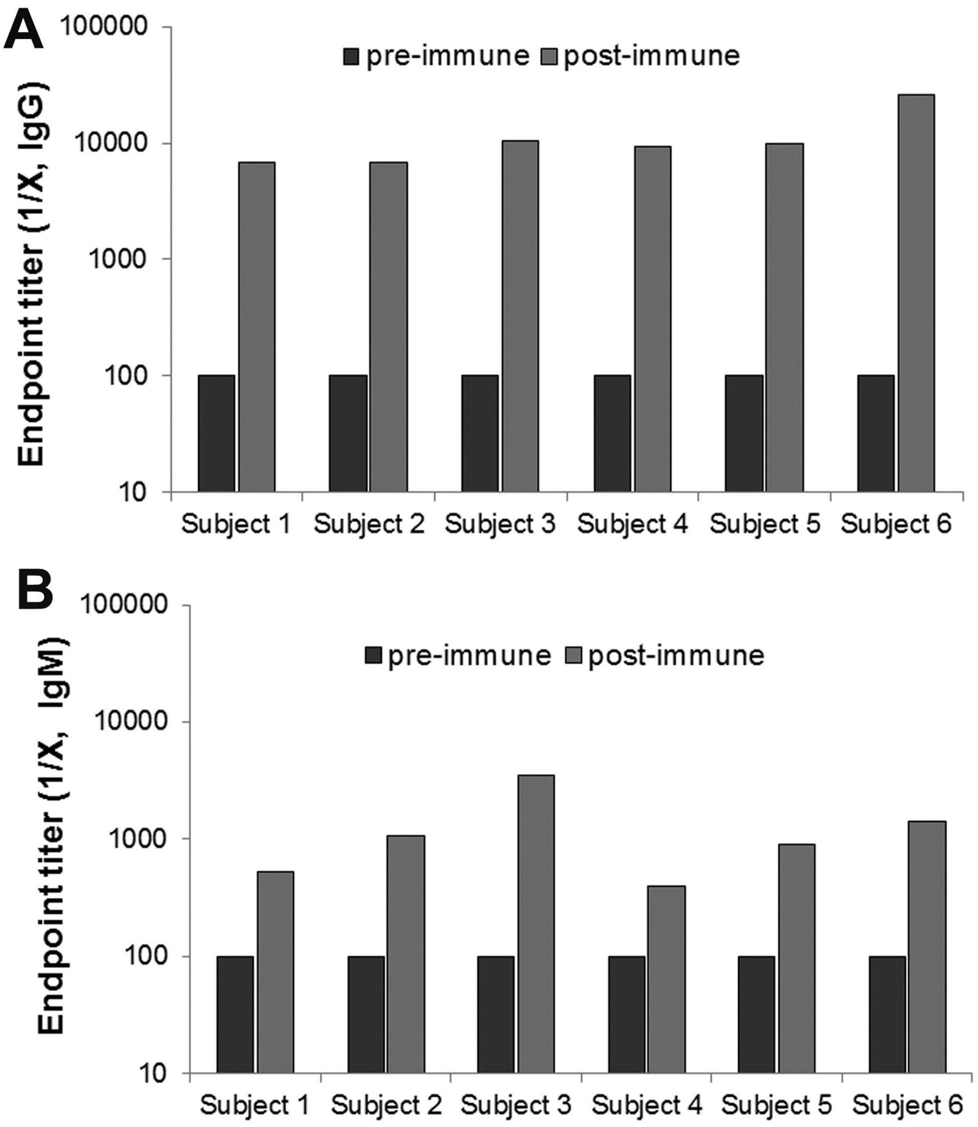

Supplementary Figure 1: Reactivity of Subject plasma antibodies against P10s. ELISA plates were coated with multivalent antigen peptide (MAP) version of P10s and reactivity of two-fold serial dilutions of pre- (week 1) and postimmune (week 7) plasma was detected by HRP-conjugated anti-human IgG (**A**) and IgM (**B**). Normalized endpoint titers were estimated as described in the Materials and Methods section and in the legend to Figure 1.

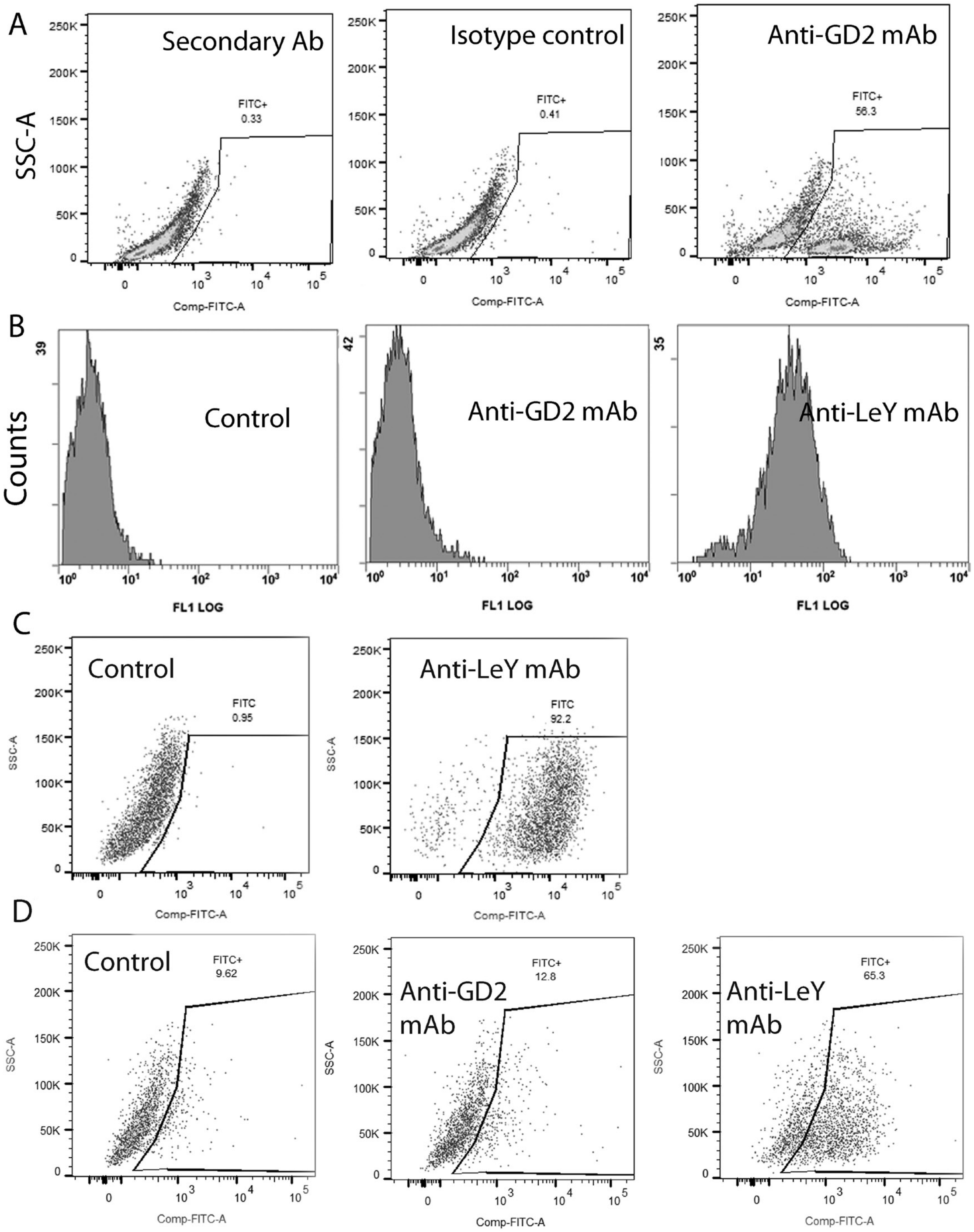

Supplementary Figure 2: Flow cytometry assay of GD2 and LeY expression on breast cancer cell lines used in this study. MDA-MB-231 (**A**), and HCC1954 (**B**) cells express GD2 and LeY antigens, respectively. LeY is highly expressed on MCF-7 (**C**) and ZR-75–1 (**D**) cell lines. Cells were stained with anti-GD2 mAb 14G2a (10 µg/ml) or anti-LeY mAb Br55-2 (10 µg/ml). FITC-conjugated anti-murine IgG was used as secondary Ab.

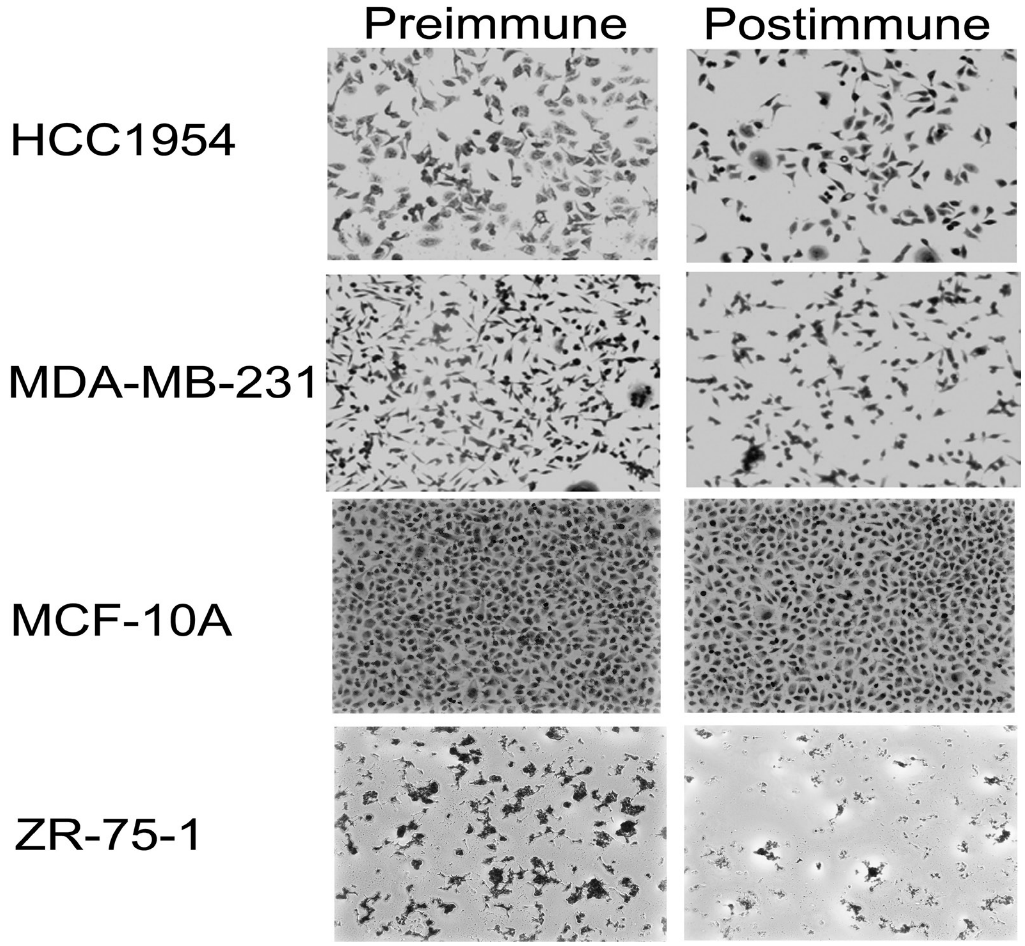

Supplementary Figure 3: Plasma antibodies from immunized subjects inhibit growth of breast cancer cell lines but no cytotoxic effect for pre- and post-immunization plasma collected from patients was observed on MCF-10A. Pre and postimmune plasma derived from subject 6 negatively affected viability of MDA-MB-231, HCC1954 and ZR-75-1 cells but did not affect viability of MCF-10A cells. Representative images taken after 24 hours of incubation with 10% plasma of the indicated subjects are shown. Alive cells were measured and the effect of pre vs. postimmune plasma on cells in three replications was quantified and illustrated in Figure 3 of the manuscript. We observe no growth inhibition effect for either pre or postimmune plasma from any subjects on MCF-10A cells – therefore no quantitation. EVOS XL cell imaging system was used to take an image from each well. A combined image file then was generated in Adobe Photoshop with individual images inserted. Extension of incubation time up to 72 hours in separate experiments did not affect viability of the MCF-10A cells.

Supplementary Table 1: Follow-up of serum endpoint titer in 4 vaccinated subjects, who were alive and had follow-up blood samples available

Year post final vaccination	Subject 3	Subject 4	Subject 5	Subject 6
Year 1	16	32	64	32
Year 2	4	2	8	4
Year 3	2	1	2	NA
Year 4	2	1	2	1
Year 5	2	1	NA	NA

NA, Not available, Titers shown as times preimmune.
